# KContact, an enhanced intervention for contact between children in out-of-home care and their parents: protocol for a cluster randomised controlled trial

**DOI:** 10.1186/s12889-015-2461-3

**Published:** 2015-11-16

**Authors:** Stephanie Taplin, Tracey Bullen, Morag McArthur, Cathy Humphreys, Margaret Kertesz, Timothy Dobbins

**Affiliations:** Institute of Child Protection Studies, Australian Catholic University, Canberra, ACT 2602 Australia; School of Social Work, University of Melbourne, Melbourne, Australia; National Drug and Alcohol Research Centre, UNSW Australia, Randwick, Australia

**Keywords:** Contact, Out-of-home care, Foster care, Intervention study

## Abstract

**Background:**

When children are unable to safely live at home with their parents, contact between these children and their parents is considered, in most cases, important for maintaining children’s sense of identity and relationships with their parents. However, the research evidence on contact is weak and provides little guidance on how to manage contact and when it is beneficial or potentially harmful. The evidence in relation to contact interventions with parents and their children who are to remain in long-term care is the most limited. A small number of studies have been identified where interventions which were therapeutic, child-focused and with clear goals, particularly aimed at preparing and supporting parents, showed some promising results. This trial aims to build on the existing evidence by trialling an enhanced model of contact in multiple sites in Australia.

**Methods/Design:**

This study is a cluster randomised controlled trial of an enhanced contact intervention with children in long-term care who are having supervised contact with their parents. Intervention sites will implement the kContact intervention that increases the preparation and support provided to parents in relation to contact. Baseline and follow-up interviews are being conducted with parents, carers and agency workers at intervention and control sites. Follow-ups interviews will assess whether there has been an increase in children’s emotional safety and a reduction in distress in response to contact visits with their parents (the primary outcome variable as measured using the Strength and Difficulties Questionnaire), improved relationships between children and their parents, improved parental ability to support contact, and fewer contact visits cancelled.

**Discussion:**

By increasing the evidence base in this area, the study aims to better guide the management and supervision of contact visits in the out-of-home care context and improve outcomes for the children and their families.

**Trial Registration:**

Trial registered on 7 April 2015 with the Australian New Zealand Clinical Trials Registry ACTRN12615000313538

## Introduction

When children are unable to safely live at home with their parents, contact between these children and their parents is considered, in the majority of cases, important for maintaining children’s sense of identity and relationships with their parents, and as a means of enhancing their emotional, behavioural and intellectual development [1, 2]. However, the research evidence on contact is weak and provides little guidance on how to manage contact, and for which children and in what circumstances it is beneficial or potentially harmful [2–4]. One the one hand, good quality contact in conjunction with other constructive professional intervention has been found to promote positive outcomes for children and is positively correlated with children’s current wellbeing and stability [1, 5]. On the other hand, contact can be disruptive: it can prevent children developing a sense of permanence, cause additional emotional strain on children, and can increase conflicts between parents, carers and children [1, 6, 7]. It is important, therefore, that contact service providers and agencies with the parental responsibility for children in the care of the state minimise the possibility that these children will experience further distress as a result of their family contact visits [8]. Neither should contact contribute to further adverse outcomes for their mothers and fathers which may, in turn, lead to a loss of contact with their children. Designing and supporting contact interventions that improve outcomes for children and their families is therefore vital.

The study discussed in this paper was designed to address gaps in the research evidence by developing and trialling an evidence-informed model of delivering contact. The focus of this study is on supervised contact for children aged 0 to 14 years in long-term foster or kinship care.

## Background

Direct contact (the focus of this research) refers to the planned face-to-face visits with parents or significant others when parents are no longer providing primary care to the child or young person [9]. Contact may also be referred to in the literature as ‘access’ or ‘visitation’. The legislation governing the care and protection of children in Australia supports continued contact between children in care and their parents, as does the *United Nations Convention of the Rights of the Child*, Article 9 [10] which supports the rights of children to maintain personal relationships with their parents, unless this would not be in the best interests of the child.

Contact may be supervised if there are particular concerns about the ongoing safety of the child and the type of abuse that led to the child being removed [11, 12]. Other factors such as the age of the child and their relationship with the parents may also be influential in making decisions about supervision [1]. Over time the need for and appropriateness of supervision may change, however, as the placement becomes established and the child’s age increases and their vulnerability decreases [8].

Supervised contact refers to contact in which interactions and conversations between the parent and child are closely monitored by a nominated person, such as a relative or carer, or via an agency such as a contact service [13]. Some authors have noted that there is no common understanding of the concept, definition, or purpose of supervised contact amongst service providers working in the child protection system [14]. Formal contact supervisors may either be there to simply observe and take notes about the interactions between parent and child, while ensuring safety, or they may have a more engaged role that supports and enhances parent–child interactions [4]. Supervised contact visits have been described as providing a therapeutic experience, an evaluative method for assessing parental bonding, or a proactive method of enhancing poor parenting skills [8, 15]. Currently there are no coherent and empirically-based theory and guidelines with which to judge the quality of contact, nor criteria for evaluating what occurs during meetings between children and their parents [4].

It has been reported in the literature that between 56 % and 94 % of children in foster and kinship care have some direct contact with their parents, although this generally reduces over time [2, 16–18]. A small number of studies have reported that around 50 % of children in kinship care have this contact supervised, while higher proportions of children in foster care have their contact supervised (56-67 %) [2, 17, 18].

### Contact interventions

The few contact interventions identifiable in the literature tend to focus on contact for families for whom reunification of the child and their parents is the goal. The evidence in relation to contact interventions with parents and their children who are to remain in care is more limited. A small number of studies have been identified where interventions which were therapeutic, child-focused and with clear goals showed some promising results. These interventions included components such as: clarifying the purpose of contact visits; providing greater support and structure to parents, children, caseworkers and carers; and facilitating, planning and increasing the preparation for all of those involved [19–22]. Findings from qualitative studies indicate that support via training and/or emotional support, facilitating planning, and enhancing communication for all those involved in providing family contact are valued and may lead to higher quality contact [23–25].

This paper describes the design and protocol for the kContact study, a cluster randomised controlled trial being conducted in Victoria and the Australian Capital Territory (ACT), Australia. The kContact intervention developed in the first stage of this study will be trialled using a prospective design that compares (i) children and young people who receive treatment as usual (standard contact) with (ii) children and young people receiving the kContact intervention, at baseline and nine months. It will be innovative for the child welfare field in its use of both qualitative and quantitative components and its use of multiple study informants (experts, children, parents, carers, workers and the study’s partner organisations delivering the contact services).

### Aim and hypotheses

The aim of the trial is to test the effectiveness of an enhanced model of managing contact for children in long term out-of-home care and their parents (the kContact intervention).

It is hypothesised the intervention will:

i) increase children’s emotional safety and reduce their distress related to contact (primary outcome variable);
ii) improve relationships between children and their parents;
iii) improve the ability of parents to support children in the context of contact visits;
iv) reduce the proportion of contact visits cancelled within the nine month follow-up period in comparison to a control group.

## Study Design

### Trial registration

This trial was registered with the Australian and New Zealand Clinical Trials Registry (ACTRN12615000313538)

### Ethics approval

Ethics approval was obtained from the Australian Catholic University’s Human Research Ethics Committee (HREC), and ratified by the University of Melbourne HREC. Approvals were also obtained from the Victorian Government Department of Health and Human Services (DHHS), ACT Community Services Directorate and our non-government partner agencies to conduct the study within their organisations.

### Recruitment of agencies and programs

Ten government and non-government agencies providing foster and kinship care services, which included the supervision of contact for children in care, in either ACT or Victoria agreed to partner with the researchers in contributing towards and applying for research funds to conduct the kContact study. All agencies had agreed to being randomised to either the intervention or control group.

Because of the variation in agency size, the number of locations and range of programs (foster and kinship care) provided by some of these agencies, the ten agencies were divided into 18 programs for the purposes of the study: these 18 programs form the study clusters.

### Randomisation of programs (clusters)

Allocation of the programs (clusters) was undertaken using the following randomisation process. Meetings were held in both jurisdictions with representatives of all the participating agencies present, to ensure the transparency of the process. The program sites were paired according to the size of the program. To minimise selection bias, for each of the nine pairs an independent researcher randomly selected each site (using opaque, identical, sealed envelopes) to either the intervention group or the control group, by picking envelopes out of a box. This resulted in the selection of nine intervention and nine control sites across the two jurisdictions.

As is standard in a cluster randomised trial, the intervention will be implemented across the intervention sites and all clients of that program will receive the intervention whether they are actively recruited into the study or not. Staff involved in providing supervised contact for children and their parents in the intervention sites will be provided with specific training, support, a manual and resources by the kContact team. The control group sites will continue to provide supervised contact services to children and their parents as outlined in their case management plan and their agreed contact arrangements, or “treatment as usual”. Control group sites will be provided with training and resources to adopt the intervention, should they wish to, at the conclusion of the study.

### Blinding (allocation concealment)

This is an open, unmasked study. Steps have been taken, however, to ensure the separation and independence of the outcome data collection from the support of the intervention, by ensuring that the research team member providing the intervention training and monitoring the fidelity of the intervention does not collect outcome data.

### Sample size

There are no estimates of effect size on which to base our sample size calculations, as there are no trials of contact interventions published. We have made some assumptions to determine the sample size necessary to detect change in the primary outcome variables (namely, increase children’s emotional safety and reduction in their distress related to contact) using PASS software (2014). As there are no estimates available for the matching correlation, we have used a conservative approach and estimated power using an unmatched cluster randomised design [26]. Assuming an intracluster correlation coefficient of 0.06 [27] and an average cluster size of 10, randomising 18 clusters to two groups will yield 100 % power to estimate an effect size of 1.0; 72 % power to estimate an effect size of 0.5; and 39 % power to estimate an effect size of 0.33.

### Selection criteria for participants

Study participants are being recruited from the participating study sites, both intervention and control. Because the study intervention is focused on supervised contact between children in long-term care and their parents, the selection criteria developed was as follows: children aged up to 14 years in long-term care (on long-term care orders) having supervised contact with one or more parent which is managed by one of the participating agencies.

Although the major study outcomes are in reference to the study child, much of the data will be collected from other informants (namely, carers, parents and caseworkers). However, as it is important to include the voices of children about issues that affect them, as part of this study, children aged 8–14 years are being interviewed but only in the ACT as part of a PhD study. This age group was selected based on a view that 8 years is the minimum recommended age for interviewing children in the child protection system [28].

### Procedure for the identification of eligible participants

The procedure used to identify children who fit the selection criteria is as follows: participating agencies plus the ACT and Victorian government agencies developed lists of individual eligible children and their families (children aged 0–14 years in long–term out-of-home care having supervised contact through a partner agency) which were provided to the researchers, to be stored securely. To avoid duplication, only one child per family is recruited, unless two children are having separate contact with each parent. Where there is more than one eligible child having supervised contact with one parent, simple randomisation using a computer-generated randomisation table is used to select the study child.

The ACT child protection agency, who is a partner on the project, provided signed consent forms to the researchers which gives permission for carers and agency workers to be interviewed in relation to the eligible children, for children aged 8 to 14 years to be interviewed in ACT, and to provide information that will help locate the child participants for follow-up interviews. It was a requirement of the child protection department (DHHS) in Victoria that consent forms be signed by parents who have shared parental responsibility with the Department.

### Recruitment of participants

To engage the study sites, researchers attended a series of meetings and information sessions with agency workers to outline the study rationale, plan and recruitment processes. Letters were also sent to eligible carers and parents, information was provided in agency newsletters, and flyers were distributed throughout the agencies to increase knowledge and awareness of the study.

Recruitment of individual participants (parents, carers and caseworkers) is undertaken as follows: potential participants are first approached by the agency staff representative who provides a brief overview of the study. Potential participants are given the option of either directly contacting the research team (by phone or email) or providing their contact details to agency staff so the researchers can contact them to discuss the study in greater detail. If the potential participant agrees, an appointment is made and they are taken through an Informed Consent process.

In addition, in the ACT carers are asked to contact the doctoral student who is interviewing eligible children aged 8–14 years, and who undertakes a separate Informed Consent process, to be described in a separate paper.

Baseline interviews are being conducted between March and October 2015, prior to the implementation of the intervention in the intervention sites. Follow-up interviews will be conducted approximately nine months after the baseline interviews.

### Data collection procedure

Interviews with workers, parents and carers are conducted by a kContact researcher in person in a location convenient to the participants, which ensures both the privacy and the safety of all parties. Interviews are 60 to 90 minutes in duration. Although face-to-face interviews are the preferred method, data collection methods may be varied, particularly taking into account the choice of the participant and their location, to include telephone interviews.

Interview schedules designed specifically for the study include a number of open-ended questions. Valid and reliable scales are being used to collect information in relation to the outcome variables. Interview schedules were piloted with non-eligible participants prior to study commencement, which resulted in some minor changes to question wording. The standardized scales may be self-completed by the research participant should they choose to do so.

Similar questions are being used with all participant groups (parents, carers and caseworkers), to facilitate comparisons between groups, and at the baseline and follow-up interviews. Further discussion of the outcome measures is provided in subsequent sections.

At the conclusion of their interview, parents are asked to provide the contact details of two people who will know their location at the time of the follow-up interviews, to increase the likelihood that the researchers will be able to re-contact the parents. This identifying information is stored separately and securely from their response information.

Carers and parents are both provided with a AU$50 voucher at the conclusion of each interview in recognition of their contribution to the research.

### Development of the intervention model

The main aim of this study is to develop and trial an evidence-informed model of delivering supervised contact. The methodology used to develop the intervention model is based on a methodology developed in the mental health field to develop treatment “outlines” for psychiatric disorders [29]. This methodology involved a comprehensive review of the literature, consultations and interviews with stakeholders, workshops with agency staff and practitioners, and a consultation with an expert panel, all of which informed the intervention developed.

Initial consultations were undertaken with a range of stakeholders who managed, delivered or made decisions about ‘contact’ to identify the major gaps and issues in the delivery of supervised contact services. Those consulted included Children’s Court magistrates, policy-makers, experts in ‘contact’, psychologists, parents, carers, children in out-of-home care and contact service providers.

Following these consultations, a comprehensive review of the published and grey literature was undertaken, to be described elsewhere. From this literature review, two key models of supervised contact for children in care were identified which demonstrated some promising preliminary findings in relation to improved outcomes for children and which were feasible in the context of this study. These models were: (i) “Visit Coaching”[20, 21] and (ii) a strengths-based approach to supervised visits [22]. Shapiro and Sims (2014) have presented preliminary results from their strengths-based intervention that showed some improvements in parents’ attitudes and skills, along with some increase in the children’s levels of resilience, as measured by the Devereux childhood assessment scales [22, 30]. Pilot trial data of the Visit Coaching model suggests that reunifications were more successful, that parents reported increased transparency in communication with the agency, and that it facilitated stronger relationships with foster carers in comparison to other models [31] Both of these models target the level of support for parents around contact with the goal of increasing their parenting skills and improving their ability to relate to their children at contact visits. These two models form the foundation of the kContact intervention model.

Workshops were then conducted with contact agency staff and policy-makers. At these workshops the findings from the literature review were presented and discussed, and the proposed focus and direction for the contact intervention was ratified by the attendees. Once the literature review was refined and a draft outline of the intervention developed, a further workshop with an expert panel consisting of three external experts in contact was held. This expert panel confirmed the proposed intervention to be trialled.

Many factors influence contact visits themselves which are outside the control of the research team, such as location and frequency of visits, and consistency of the contact supervisor. Furthermore, the contact visit itself is regulated by case planning and Children’s Court decisions. The researchers, in consultation with the expert panel, therefore decided to limit the kContact intervention to an enhancement of current practice in relation to contact by providing support outside visits only, rather than during contact visits.

### The kContact intervention model

The kContact intervention consists of structured support provided to parents both prior to and following supervised contact visits with their child(ren) in long-term out-of-home care. Drawing on the literature and the input from consultations, a kContact Intervention Manual was developed by the research team in conjunction with an expert in communications and training, the kContact Intervention Coordinator. All the information needed to deliver the intervention is described in the kContact Intervention Manual, which will be made available to agency staff in the intervention sites only. The Manual describes the four stages of the intervention (excluding the contact visit) in detail.

It is intended that each of the four intervention components will be of no more than 15 minutes in duration, to limit the burden on agencies, but may be varied depending upon the parents’ needs at the time. The intervention is to be delivered by the key worker, who is the practitioner at the intervention site (usually a caseworker or contact worker) who has an existing relationship with the parent or who is best placed to develop this relationship and support the intervention; they may or may not be the supervisor of the actual contact visit.

The kContact intervention stages are as follows:

i. The planning component consists of: providing an overview of the Intervention, discussing expectations and concerns, confirmation of attendance at visits, and an assessment and discussion of the children’s needs to be met during visits, based on parents’ knowledge and experience of their children developmentally.
ii. The preparation or pre-visit planning component involves identifying the goals and aims they would like to achieve during visits with their children and jointly planning activities for the contact visit to reflect these goals, as well as communication of relevant information to parents before the visit.
iii. The supervised contact visit (kContact has no direct input into this stage).
iv. The follow-up visit component involves encouraging parents to reflect on what worked well, with an emphasis on the strengths they could build on, validating parents’ feelings about the visit, including feelings of grief, distress or anger, and discussing aspects of visits that could be managed differently at subsequent visits.
v. Lastly, the review component involves a review of the broader goals of visits and the progress towards those goals from the point of view of children, parents, carers and relevant professionals.

Each component has optional additional resources that can be provided to parents and used by the key worker to facilitate planning and further discussions.

### Intervention training and support

After the completion of the baseline interviews, agency staff members in each kContact intervention site will be booked to attend a half day training session on the conduct of the intervention. The training session consists of an outline of: (i) the rationale and development of the intervention; (ii) an overview of and structure of the intervention; (iii) a discussion of any staff concerns about the implementation and delivery of the intervention; and (iv) the provision of suggestions as to how to best deliver the intervention to parents, using case scenarios. The kContact Intervention Coordinator is responsible for conducting the training sessions, and will also be available for consultation and support following the training to address any difficulties with implementation and delivery of the intervention.

### Intervention fidelity

Intervention fidelity refers to the extent to which core components of interventions are delivered as intended by the protocols; ensuring and measuring fidelity is important in detecting the effects of the intervention [32]. In the present study, a number of steps will be implemented to facilitate fidelity. Key workers will be provided with an Intervention Checklist to remind them of the stages of the intervention and to assist the research team to monitor its delivery. Also at intervention sites, caseworkers will be asked to either audio-record a sample of their sessions with parents to be forwarded to the researchers, or have their manager supervise and record their observations of a sample of sessions via a manager’s checklist, also to be returned to the researchers.

### Outcome measures

The major primary and secondary outcome variables were identified from the literature and are outlined in Table 1. They are being measured via the use of valid and reliable scales where they are relevant and a suitable measure exists. Where possible, measures used in similar Australian and international studies are used to enable comparisons between studies such as the Longitudinal Study of Australian Children (LSAC) and the Pathways of Care Longitudinal Study (POCLS) [33]. Both primary and secondary outcomes will be assessed at baseline (prior to the intervention delivery) and at follow-up, approximately nine months later, thereby allowing any change that can be attributed to the intervention to be detected. In addition, background information will be collected from carers (about their training and experience), parents (regarding their characteristics and risk factors) and from workers (about their professional background and the background of the children, such as the children’s care history). Information will also be collected from each respondent about their views and experiences of contact visits. Much of this information is collected via questions designed specifically for the study.

**Table 1:** Outcomes, measures and interviewees providing the information

Outcomes	Outcome measures	Interviewee		
Primary outcome		Carer	Parent	Worker
Psychological distress and emotional safety (child)	Strength and Difficulties Questionnaire (SDQ)	✓		
Secondary Outcomes				
Quality of relationships between children, parents and carers	Child Parent Relationship Scale Short Form (CPRS-SF)	✓	✓	
Quality of relationships between children, parents and carers	NSW Pathways of Care Longitudinal Study questions	✓		✓
Quality of relationships between children, parents and carers	Relatedness Scale			
Ability of carers to support children around contact	Receptivity to Birth Family Connections Scale (RBFCS)	✓		✓
Abuse potential	Brief Child Abuse Potential Inventory (BCAP)		✓	
Adult distress	Depression Anxiety Stress Scale 21 (DASS-21)	✓	✓	
Satisfaction with contact	Adapted from Salveron et al. (2009)		✓	
Proportion of contact visits cancelled	Self-report questions designed for study; administrative data	✓	✓	✓

### Primary outcome

Child distress and a lack of emotional safety have been identified as potential negative outcomes from contact visits, and a number of researchers have recommended that contact services minimise the possibility that children will experience further distress as a result of their family contact visits [8]. Children’s emotional safety and distress will be the primary outcomes variable in this study, measured using the Strengths and Difficulties Questionnaire (SDQ), a widely-used scale which assesses levels of internalising and externalising psychosocial problems and prosocial behaviours [34]. Carers will complete the SDQ in relation to the study child who is in their care.

### Secondary outcomes

The secondary outcome measures proposed to detect change in response to the intervention are (i) quality of relationships between children, parents and carers, (ii) ability of carers to support children around contact, (iii) parenting capacity, and (iv) proportion of contact visits cancelled within the nine month follow-up period. The quality of relationships between children and parents and children and carers will be measured using the Child Parent Relationship Scale (CPRS) short form which assesses levels of closeness and conflict [35]. The ability of carers and workers to support contact between parents and children will be assessed using the Receptivity to Birth Family Connections Scale (RBFCS) [36]. Parenting capacity will partly be measured via the Brief Child Abuse Potential inventory (BCAP) which assesses the risk of physical abuse [37]. The Depression Anxiety Stress Scale-21 (DASS-21) will be used to measure the distress levels of parents and carers [38, 39] and has been widely used with similar populations [40, 41] and in randomised control trials [42, 43]. Other measures on parental satisfaction with contact [44] and information on cancellations of contact visits designed for the study will be obtained from all adult participants, including agency staff, to determine if parents’ attendance at contact changes in response to the intervention.

### Planned analyses

Comparisons of the control and intervention groups will be made at baseline by calculating descriptive statistics to assess randomisation. Analysis for the continuous primary outcome, SDQ, will be performed using a linear mixed model to allow for the matched, clustered design of the trial. We will adjust for the baseline value and any potential confounding variables that display baseline imbalance between intervention groups. Secondary outcomes will be analysed in a similar way. For dichotomous variables, we will use a generalised linear mixed model, which extends the standard logistic regression model to account for the clustered data. The qualitative data will be coded for themes and used to add additional context to the quantitative data.

## Discussion

This paper outlines the study protocol for the first cluster randomised control trial of a contact intervention, being conducted in ACT and Victoria, Australia. The aim of the trial is to increase children’s emotional safety and reduce their distress in response to contact visits with their parents. It also aims to improve the quality of the relationship between children and their parents by providing parents with additional support and guidance to prepare for, interact with and reflect on supervised contact visits with their children in out-of-home care. The intervention has been developed using the existing research evidence and via consultations with key stakeholders and an expert panel. By increasing the evidence base in this area, the study aims to better guide the management and supervision of contact visits in the out-of-home care context and improve outcomes for the children and their families.

## References

[1] Sen R, Broadhurst K (2011). Contact between children in out-of-home placements and their family and friends networks: a research review. Child Family Social Work.

[2] Taplin S, Mattick RP (2014). Supervised contact visits: results from a study of women in drug treatment with children in care. Child Youth Serv Rev.

[3] Quinton D, Selwyn J, Rushton A, Dance C (1999). Contact between children placed away from home and their birth parents: Ryburn's 'reanalysis' analysed. Clin Child Psychol Psychiat.

[4] Triseliotis J (2010). Contact between Looked after Children and Their Parents: A Level Playing Field?. Adoption Fostering.

[5] McWey LM, Acock A, Porter BE (2010). The impact of continued contact with biological parents upon the mental health of children in foster care. Child Youth Serv Rev.

[6] Barber JGD, Paul H (2004). Children in foster care.

[7] Morrison J, Mishna F, Cook C, Aitken G (2011). Access visits: Perceptions of child protection workers, foster parents and children who are Crown wards. Child Youth Serv Rev.

[8] Saini M, van Wert M, Gofman J (2012). Parent–child supervised visitation within child welfare and custody dispute contexts: An exploratory comparison of two distinct models of practice. Child Youth Serv Rev.

[9] Scott D, O'Neil C, Minge A (2005). Contact between children in out-of-home care and their birth families.

[10] Assembly UG (1989). Convention on the Rights of the Child. United Nations Treaty Series.

[11] Court NC: Contact guidelines. 2011.

[12] Sinclair I, Gibbs I, Wilson K (2004). Foster Carers: Why they stay and why they leave.

[13] Perry A, Rainey B (2007). Supervised, Supported and Indirect Contact Orders: Research Findings. Int J Law Policy Family.

[14] Wattenberg E, Troy K, Beuch A (2011). Protective supervision: An inquiry into the relationship between child welfare and the court system. Child Youth Serv Rev.

[15] Ellenbogen S, Wekerle C (2008). Visitation practices in child welfare organizations. J Ontario Assoc Child Aid Soc.

[16] Sinclair I, Baker C, Gibbs I, Wilson K (2005). Foster Children: Where they go and how they get on London.

[17] Farmer E, Moyers S (2008). Kinship care: Fostering effective family and friends placements.

[18] Hunt J, Waterhouse S, Lutman E (2010). Parental Contact for Children Placed in Kinship Care through Care Proceedings. Child Family Law Quarterly.

[19] Tilbury C, Osmond J (2006). Permanency planning in foster care: A research review and guidelines for practitioners. Aust Soc Work.

[20] Beyer M (2004). Visit Coaching: Building on Family Strengths to Meet Children’s Needs.

[21] Beyer M (2008). Visit Coaching: Building on Family Strengths to Meet Children's Needs. Juv Fam Court J.

[22] Smith GT, Shapiro VB, Sperry RW, LeBuffe PA (2014). A Strengths-based Approach to Supervised Visitation in Child Welfare. Child Care Prac.

[23] Osmond J, Tilbury C (2012). Permanency Planning Concepts. Child Aust.

[24] Fernandez E. Accomplishing Permanency: Reunification Pathways and Outcomes for Foster Children. New York. Springer. 2013

[25] Hojer I (2009). Birth parents' perception of sharing the care of their child with foster parents. Vulnerable Children and Youth Stud.

[26] Donner A, Klar N (2000). Design and Analysis of Cluster Randomization Trials in Health Research. London Arnold.

[27] Miller-Lewis LR, Sawyer AC, Searle AK, Mittinty MN, Sawyer MG, Lynch JW (2014). Student-teacher relationship trajectories and mental health problems in young children. BMC Psychol.

[28] Healy K, Darlington Y (2009). Service user participation in diverse child protection contexts: principles for practice. Child Family Soc Work.

[29] Project TQA (1982). A Methodology for Preparing ‘IDEAL’ Treatment Outlines in Psychiatry. Aust N Z J Psychiatry.

[30] Shapiro VBS (2014). Heidi. : A Strengths-based Approach to Supervised Visitation in Child Welfare. Evidence Informed Practice Series. California Social Work Education Center Research and Training Network.

[31] Cutshaw CA, Affronti M, Elliot SA, Beyer M (2012). Family Visit Coaching for Children in Foster Care: Setting a Research Agenda and Diving. 18th National Conference on Child Abuse and Neglect: 2012; Washington, D.C.

[32] Gearing RE, El-Bassel N, Ghesquiere A, Baldwin S, Gillies J, Ngeow E (2011). Major ingredients of fidelity: A review and scientific guide to improving quality of intervention research implementation. Clin Psychol Rev.

[33] Paxman M, Tully L, Burke S, Watson J (2013). Pathways of Care: Longitudinal study on children and young people in out-of-home care in New South Wales.

[34] Goodman R (2001). Psychometric properties of the strengths and difficulties questionnaire. J Am Acad Child Adol Psychiat.

[35] Pianta RC (1992). Child–parent relationship scale.

[36] Orme JG, Cox ME, Rhodes KW, Coakley T, Cuddeback GS, Buehler C (2006). Casey home assessment protocol (CHAP): Technical manual.

[37] Ondersma SJ, Chaffin MJ, Mullins SM, LeBreton JM (2005). A brief form of the Child Abuse Potential Inventory: Development and validation. J Clin Child Adolesc Psychol.

[38] Antony MM, Bieling PJ, Cox BJ, Enns MW, Swinson RP (1998). Psychometric properties of the 42-item and 21-item versions of the Depression Anxiety Stress Scales in clinical groups and a community sample. Psychol Assess.

[39] Lovibond S, Lovibond PF: Manual for the depression anxiety stress scales: Psychology Foundation of Australia; Sydney Australia 1996.

[40] Whenan R, Oxlad M, Lushington K (2009). Factors associated with foster carer well-being, satisfaction and intention to continue providing out-of-home care. Child Youth Serv Rev.

[41] Doley R, Bell R, Watt B, Simpson H (2015). Grandparents raising grandchildren: investigating factors associated with distress among custodial grandparent. J Family Stud.

[42] Hiscock H, Bayer JK, Price A, Ukoumunne OC, Rogers S, Wake M (2008). Universal parenting programme to prevent early childhood behavioural problems: cluster randomised trial. BMJ.

[43] Dittman CK, Farruggia SP, Palmer ML, Sanders MR, Keown LJ (2014). Predicting success in an online parenting intervention: The role of child, parent, and family factors. J Fam Psychol.

[44] Salveron M, Lewig K, Arney F (2009). Parenting groups for parents whose children are in care. Child Abuse Rev.

